# Single-cell transcriptomics uncover the key ferroptosis regulators contribute to cancer progression in head and neck squamous cell carcinoma

**DOI:** 10.3389/fmolb.2022.962742

**Published:** 2022-08-08

**Authors:** Fei Liu, Lindong Tang, Qing Li, Leihui Chen, Yuyue Pan, Zhao Yin, Jingjun He, Junzhang Tian

**Affiliations:** ^1^ Cancer Screening Center, Department of Health Management, Guangdong Second Provincial General Hospital, Guangdong, China; ^2^ Institute of Hematology School of Medicine Jinan University, Guangdong, China; ^3^ Department of Stomatology Guangdong Second Provincial General Hospital, Guangdong, China; ^4^ Department of Hematology Guangdong Second Provincial General Hospital, Guangdong, China

**Keywords:** head and neck squamous cell carcinoma, cell heterogeneity, ferroptosis, prognostic, single cell RNA sequencing

## Abstract

The mechanism underlying the association between the development of head and neck squamous cell carcinoma (HNSCC) and ferroptosis is unclear. We analyzed the transcriptomes of 5902 single cells from a single-cell RNA-sequencing (scRNA-seq) dataset. They then aggregate into B cells, epithelial cells, fibroblasts, germ cells, mesenchymal cells, cancer stem cells, stem cells, T cells and endometrial cells, respectively. Our study shows that multiple pathways are significantly enriched in HNSCC development including extracellular matrix structural components, humoral immune responses, and muscle contraction. Differentially expressed genes analysis in Pseudotime analysis, pathway and biological function indicated that there was a significant correlation in the ferroptosis pathway. Furthermore, higher ferroptosis potential index (FPI) scores were significantly associated with worse overall survival prognosis in HNSCC patients. Pseudo-temporal, survival analyses and immunohistochemistry identified multiple central genes in HNSCC development, including ACSL1, SLC39A14, TFRC, and PRNP genes, and indicated associated ferroptosis. Overall, our study detected ferroptosis-related features is closely correlated with HNSCC prognosis and development, and deserved candidates suitable for immunotherapy treatment strategies determination for HNSCC patients.

## Introduction

Head and neck squamous cell carcinoma (HNSCC), which is frequently encountered in the clinical setting, is the seventh leading cause of cancer-related death worldwide: approximately 700,000 new cases and 350,000 deaths were reported worldwide in 2018 (Membreno et al., 2021; Sacco et al., 2021). HNSCC mainly includes cancers of the nasal cavity, paranasal sinus, oral cavity, pharynx, and throat, and over 90% of these cancers are squamous cell carcinomas (Jing et al., 2021; Membreno et al., 2021). Although there have been continuous advances in the field of comprehensive surgery, radiotherapy, and chemotherapy in recent years, the 5-year survival rate of HNSCC has not significantly improved, and 30–40% of patients are likely to develop distant metastasis within 5 years (Membreno et al., 2021). Moreover, 58% of patients may have advanced disease (stage III to IV) when they are first diagnosed, and this is a great challenge to treatment (Hamman et al., 2021). In order to improve the diagnosis and treatment of this cancer, it is important to understand the cellular mechanisms involved in its progression and metastasis (Hamman et al., 2021; Membreno et al., 2021).

Ferroptosis is a newly discovered form of programmed cell death characterized by iron-dependent lethal accumulation of lipid peroxides (lipid reactive oxygen species or lipid ROS) that was recently identified as one of the mechanisms of cancer cell death in several cancers, including liver cancer, kidney cancer, bone cancer, and lung cancer (Chen et al., 2022; Peng et al., 2023). Ferroptosis occurs when the levels of lipid ROS exceed the cellular antioxidative threshold and oxidative stress overload is induced in cells (Zhang et al., 2022). Excess oxidative stress causes damage to large molecules such as proteins, nucleic acids, and lipids, and eventually leads to cell injury or death (Zhai et al., 2022). With regard to cellular morphology, ferroptosis is characterized by loss of membrane integrity with morphologically normal nuclei and shrunken mitochondria as well as thickening of the mitochondrial double membranes and rupture of the outer mitochondrial membrane (Zhang et al., 2022).

Ferroptosis was first discovered in the study of the lethal mechanisms of the small molecule drug erastin against tumor cells carrying a mutation of the oncogene RAS (Liang et al., 2019). Erastin can bind to voltage-dependent anion channel-2/3 of mitochondria to induce ferroptosis of cancer cells. Erastin can also inhibit the function of the cystine/glutamate antiporter system to reduce intracellular glutathione levels, resulting in the accumulation of lipid ROS to induce ferroptosis. In addition, a series of artificial compounds, such as RSL3, DPI7, DPI10, DPI12, and DPI13, can inhibit glutathione peroxidase-4, and thereby increase the levels of peroxides in cancer cells, leading to Fe^2+^-dependent metabolic abnormalities (Yang and Stockwell, 2016). Translational research has shown that the chemotherapeutic drug sorafenib can also suppress the cystine/glutamate antiporter system to trigger cell ferroptosis in several cancers (Cao and Dixon, 2016). Furthermore, low-dose sorafenib can induce ferroptosis, but high-dose sorafenib can induce not only ferroptosis, but also other forms of programmed cell death (Peng et al., 2023). Xie et al. (2022), investigating the transcriptome data of TCGA and Chinese Glioma Genome Atlas (CGGA) database, and thus identifying 36 radiosensitivity- and 19 ferroptosis associated differentially expressed genes with a prognostic value. In results, they also revealed that the radiosensitivity- and ferroptosis-associated biomarkers, includes HSPB1, STAT3, CA9, MAP1LC3A, MAPK1, ZEB1, and TNFAIP3, with a prognostic value for gliomas patients. Huang et al. (2022), found that the ferroptosis therapy can be a effectively approach to trigger the cancer cell death, and thus activating the cell oxidative phosphorylases and promoting cell oxidative damage. Magesh and Cai (Forthcoming 2022), revealed that the ferritin play an important susceptor for abnormal mitochondrial function, metabolism and oxidative phosphorylation in tumor cells, and also a target for tumor therapy.

As explained above, there is evidence to demonstrate the role and mechanisms of ferroptosis in several cancers, but the mechanism of the association among HNSCC development and ferroptosis is unclear. In this article, we integrated the single-cell sequencing data and TCGA transcriptome data of HNSCC in order to systematically analyze the relationship between ferroptosis and the prognosis of HNSCC, as well as the related molecular mechanism networks and regulatory molecules, in order to identify markers for targeted treatment of HNSCC.

## Materials and methods

### Single-cell RNA sequencing analysis

**FIGURE 1:**
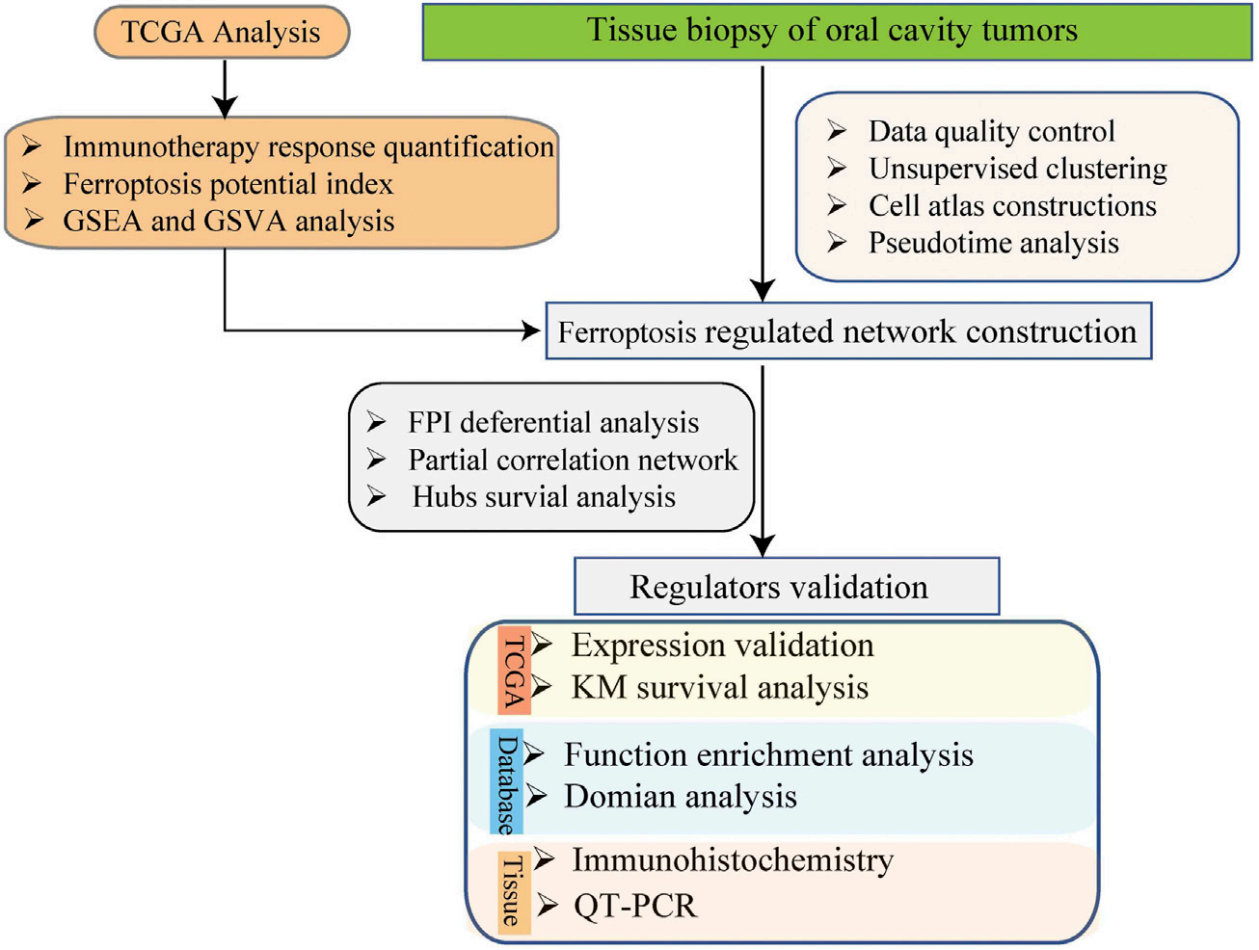
The schema of the study analysis.

The expression profiling data of 5902 single cells from the tumors of 18 patients with oral cavity tumors were downloaded from the Gene Expression Omnibus database (accession number GSE103322) (Puram et al., 2017). The expression was analyzed with single-cell RNA sequencing (scRNA-seq) using the high-throughput sequencing method Smart-Seq2. The data analysis process is described below. The flow chart of this study analysis shown in Figure 1.

1) Quality control of data: The following data filtering parameters were set: minimum number of cells = 3; minimum number of features = 200; mitochondrial gene proportion <0.05; gene count = 200–20,000 (Aran et al., 2019; Kobak and Berens, 2019).
2) Unsupervised clustering and construction of a cell Atlas: 1) normalization of the data; 2) screening of genes with high expression variation based on the mean value algorithm; 3) assessment of batch effects across samples by principal component analysis; 4) use of random sampling to construct the background distribution of correlation values between feature genes and principal components in Seurat, and use of the Jackstraw algorithm to select suitable principal components for subsequent cell cluster analysis; 5) use of the k-nearest neighbors algorithm to transform the expression profiles of cells into highly related cell clusters and identify the clusters (resolution = 0.6); 6) selection of genes that exhibit certain log-fold changes and can be used as markers in most cells based on the following criteria: min.pct = 0.25, logfc.threshold = 0.25 (Kobak and Berens, 2019).
3) Cell cluster definition: SingleR and scCATCH were used to annotate cell clusters (Aran et al., 2019).

### Pseudotime analysis

The Monocle algorithm was used to perform developmental trajectory analysis based on highly variable gene sets, and genes with high expression variation in the trajectory were selected for subsequent analysis. The criteria settings were as follows: status = ok; family = tobit; q-value < 0.05; ordering = true (Qiu et al., 2017).

### The quantification of immunotherapy response

To calculate the immunophenotypes of tumor-immune cell interactions and the cancer antigenomes, and thus predict the tumor immunotherapy response, the algorithm of Immunophenoscore was used. Here, basing the Immunophenogram algorithm, the weighted averaged Z score were identified, and shown a good predictor of response to immunotherapy, includes anti-cytotoxic T lymphocyte antigen-4 (CTLA-4) and anti-programmed cell death protein 1 (anti-PD-1) antibodies. In addition, the sum of Immunophenoscore (IPS) were calculated by histocompatibility complex (MHC)-related molecules, checkpoints or immunomodulators, effector cells, and suppressor cells (Charoentong et al., 2017).

### Correlation analysis of ferroptosis and immunotherapy response

In order to identify the immunotherapy response that were closely associated with ferroptosis, the normalized GSVA scores for ferroptosis and IPS were computed and subjected to Spearman correlation analysis to detect unsupervised clustering (Liberzon et al., 2011; Hänzelmann et al., 2013; Ferreira et al., 2021).

### TCGA analysis

The RNA-seq Recompute TPM data, clinical data, and survival data of the GDC TCGA-HNSCC datasets were downloaded from the Xena database at https://xenabrowser.net/datapages/. The DESeq algorithm was used to normalize gene expression profiles and filter out low-expression genes. The criteria for selecting differentially expressed genes were as follows: log2 |fold change|≥1.0; Benjamini-Hochberg (B-H) adjusted *p*-value < 0.05 (Anders and Huber, 2010). While the multiple comparisons were tested by one-way ANOVA, and the Bonferroni adjusted *p*-value < 0.05 was considered as significant.

### Hub gene identification and functional enrichment analysis

Hub genes were identified based on the intersection of pseudotime analysis-derived differential genes, TCGA-derived differential genes, and ferroptosis pathway genes. Next, functional enrichment analysis and binding protein prediction were performed on the identified hub genes that were deposited in the Toppgene database at https://toppgene.cchmc.org/. The filtering criterion was a false discovery rate-adjusted *p*-value of <0.05 (Chen et al., 2009; Liberzon et al., 2011).

### Calculation of ferroptosis potential index and survival analysis

Based on Liu et al.’s research, 24 ferroptosis regulator genes were selected and were classified into 1) positive regulators (LPCAT3, ACSL4, NCOA4, ALOX15, NFE2L2, NOX1, NOX3, NOX4, NOX5, GPX4, SLC3A2, and SLC7A11) and 2) negative regulators (FDFT1, HMGCR, COQ10A, and COQ10B) Liu et al. (2020). The enrichment score (ES) for the positive regulators and the negative regulators was calculated by single-sample gene set enrichment analysis (ssGSEA) in the GSVA package, and ferroptosis potential index (FPI) was calculated as the difference between the two scores. In addition, the survminer package was employed to analyze the relationship of FPI with overall survival (OS), progression-free interval (PFI), and disease-specific survival (DSS).

### Pathway enrichment analysis and GSVA

The clusterProfiler package was used to perform gene ontology (GO) analysis, Kyoto Encyclopedia of Genes and Genomes (KEGG) pathway analysis, and reactome pathway analysis on the differentially expressed genes and hub genes that were identified through pseudotime analysis. B-H adjustment (adjusted *p*-value, <0.05) was applied to differential pathways. In addition, C2 (curated gene sets), C5 (ontology gene sets), and H (hallmark gene sets) were downloaded from MSigDB at https://www.gsea-msigdb.org/gsea/msigdb/, and the GSVA and the GSEABase packages were used for standardized scoring of the gene sets for each cell (Liberzon et al., 2011). The B-H adjusted *p*-value < 0.05 were considered as significant terms in GSVA analysis.

### Immunohistochemical staining

All three HNSCC samples and paired non-neoplastic tissues used in immunohistochemical were retrieved from the Department of Pathology, Guangdong Second Provincial General Hospital, China. Before they were used, all cases were diagnosed by three certifificated pathologists without discrepancy. This research was conducted under the approval and supervision of the Ethics Committee of Guangdong Second Provincial General Hospital. Subsequently, deparaffifinized sections were treated with 3% H2O2 and subjected to antigen retrieval by citric acid (pH6.0). After overnight incubation with primary antibody (anti-ACSL1, anti-SLC39A14, anti-TFRC and PRNP antibody (Proteintech Group, China) by 1:200 at 4°C, sections were incubated for 15 min at room temperature with horseradish peroxidase-labeled polymer conjugated with secondary antibody (MaxVision Kits) and incubated for 1 min with diaminobenzidine. The sections were then lightly counterstained with hematoxylin. The sections without primary antibody were served as negative controls. Expression area of ACSL1, SLC39A14, TFRC and PRNP was determined according to the Image J (https://imagej.net/software/fiji/downloads; Life-Line versions).

## Results

### Single-cell RNA-sequencing analysis

**FIGURE 2:**
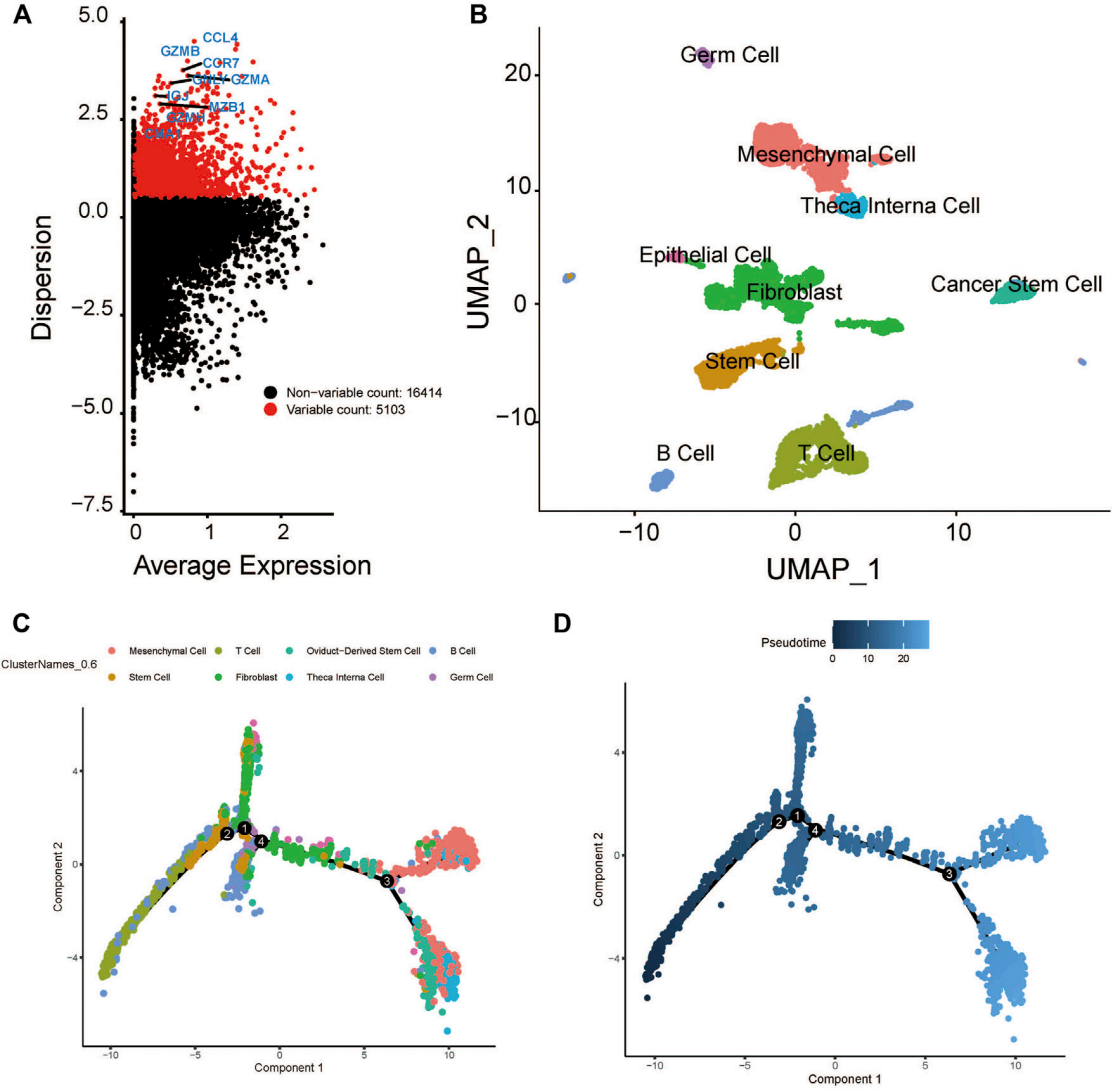
Cluster identification and pseudotime analysis of the HNSCC scRNA dataset. **(A)** Gene variation plot of the dataset, where each dot is a gene and the red dots represent genes with variable expression. **(B)** UMAP plot of HNSCC cell clusters, which are annotated as B cells, epithelial cells, fibroblast, germ cells, mesenchymal cells, cancer stem cells, stem cells, T cells, and theca interna cells. **(C)** Trajectory plot of the dataset, where each cluster (represented by each of the dots) is indicated by a different color. **(D)** Trajectory plot of the dataset, where the color of the dot indicates the differentiation state. The darker dot indicate a lower level of differential expression.

After quality control, the single-cell transcriptomic data of 5902 cells from 18 patients with oral cavity tumors were obtained. Unsupervised clustering was performed on merged datasets after normalization and correction. Cells were annotated with marker genes of known cell types. A total of 5103 genes with variable expression were identified, including GZMB, GNLY, MZB1, CCR7, and CCL4 (Figure 2A). Using t-distributed stochastic neighbor embedding and uniform manifold approximation and projection for dimension reduction, 5902 cell clusters were identified, including B cells, epithelial cells, fibroblasts, germ cells, mesenchymal cells, cancer stem cells, stem cells, T cells, and theca interna cells. The distribution of cells was plotted in a two-dimensional space (Figure 2B).

## Pseudotime analysis and enrichment analysis

The expression profiles of cells were projected onto a low-dimensional space to construct a differentiation trajectory of cells, wherein cells of similar states were aggregated together. There were four branching points along the differentiation trajectory of HNSCC that represented potential decision points of the cellular biological process (Figures 2C,D).

**FIGURE 3:**
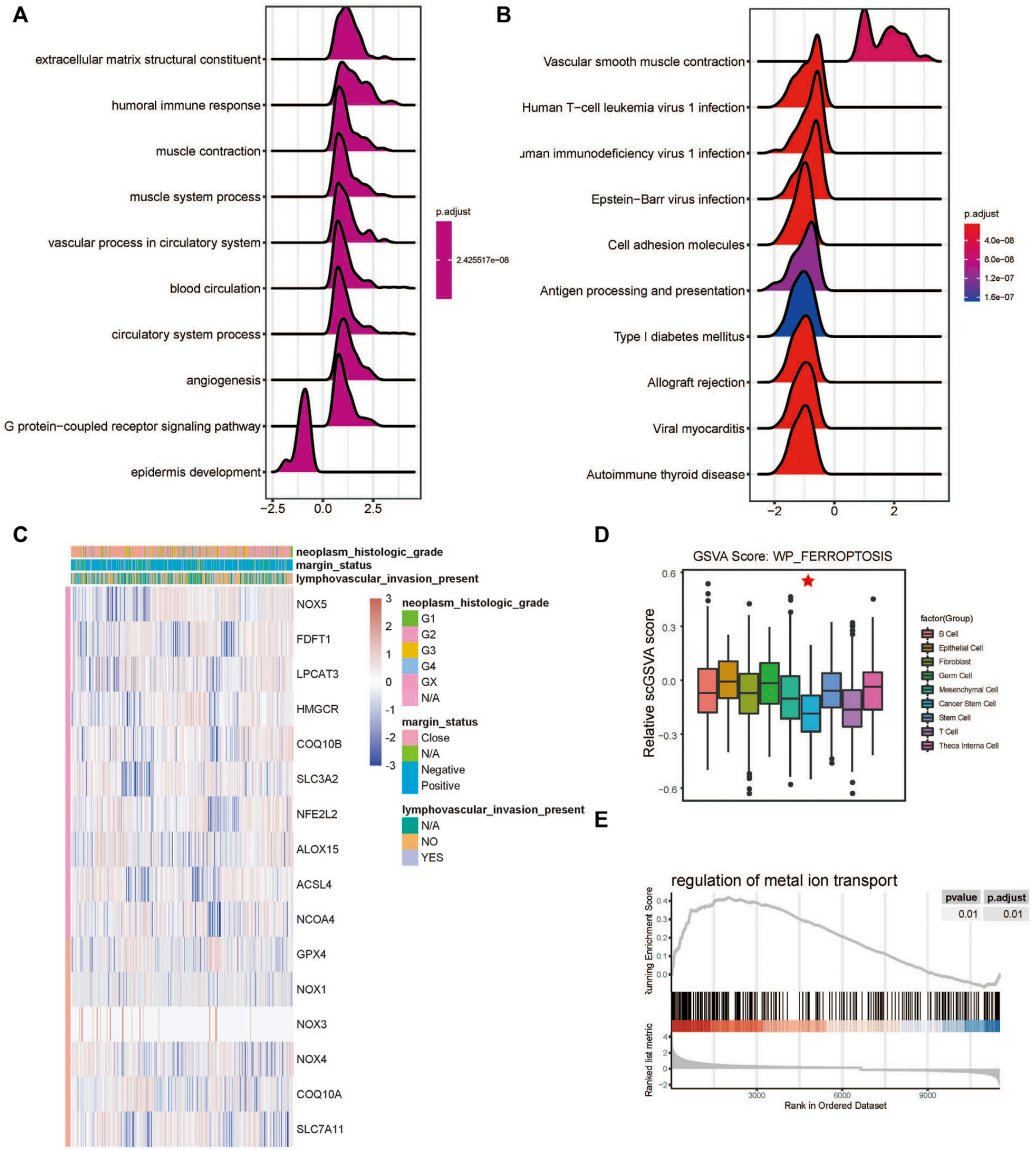
Ferroptosis-related cell cluster and pathway analysis in HNSCC. **(A)** GO and **(B)** KEGG pathway enrichment analyses of differential gene clusters from the scRNA dataset: the abscissa represents the normalized enrichment score, and the adjusted *p*-value is represented by the colored areas of the graph. **(C)** Unsupervised clustering of expression level of genes of WP-FERROPTOSIS pathway. **(D)** Distribution of WP-FERROPTOSIS score in HNSCC cell clusters. **(E)** Significant enrichment of regulation of the metal ion transport pathway in the differentially expressed genes of the cancer stem cell cluster.

The developmental trajectory analysis was performed on highly variable gene sets. Following log normalization and dimension reduction using the DDRTree algorithm, differentially regulated genes, after B-H adjustment in the pseudotime analysis, were selected for subsequent functional enrichment analyses. GO analysis showed that the following pathways were associated with the differentiation and progression of HNSCC: extracellular matrix structural constituent (ES = 0.56, normalized ES [NES] = 2.61, P.adjust = 1.00E-10), humoral immune response (ES = 0.54, NES = 2.71, P.adjust = 1.00E-10), and muscle contraction (ES = 0.51, NES = 2.69, P.adjust = 1.00E-10) (Figure 3A). KEGG analysis revealed that the following pathways were significantly enriched: vascular smooth muscle contraction (ES = 0.55, NES = 2.58, P.adjust = 1.44E-9), human T-cell leukemia virus one infection (ES = −0.34, NES = −2.52, P.adjust = 1.00E-10), and human immunodeficiency virus one infection (ES = −0.35, NES = −2.64, P.adjust = 1.00E-10) (Figure 3B). In addition, we draw a heatmap prototype for showing the expression level of genes in ferroptosis pathway (Figure 3C). Among all the cell clusters of HNSCC, the cancer stem cell cluster showed the lowest expression and highest variation in the FERROPTOSIS pathway and, therefore, was considered as the FERROPTOSIS-related core cell cluster (Figure 2D). Further, the regulation of the metal ion transport pathway was significantly enriched in the differentially expressed genes of the cancer stem cell cluster (ES = 0.42, NES = 2.24, P.adjust = 5.97E-10) (Figure 3E).

### Ferroptosis potential index and survival analysis and identification of hub genes

**FIGURE 4:**
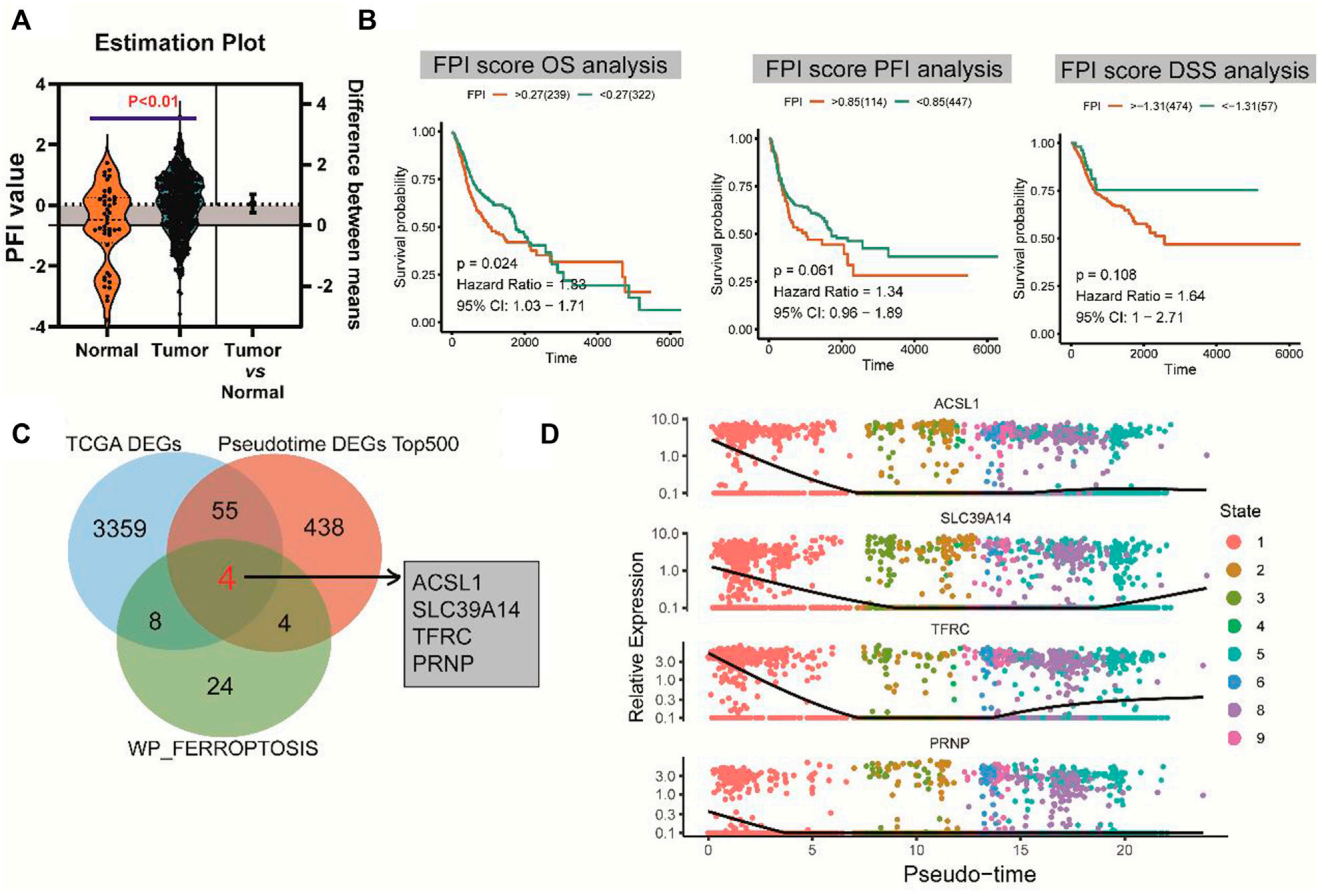
FPI and survival analysis and identification of hub genes. **(A)** Significant difference in FPI between HNSCC tumor tissues and normal tissues in TCGA. **(B)** Significant association of FPI with the OS of patients with HNSCC, but not with PFI or DSS. **(C)** Four differentially co-expressed genes or hub genes based on the intersection between pseudotime analysis-derived differential genes, TCGA-derived differential genes, and ferroptosis pathway genes. **(D)** Heatmaps of expression changes of the four hub genes from the early (left) to late stage (right).

FPI was calculated for each HNSCC sample in the TCGA database based on the method described by Liu et al. (2022). There was a significant difference in FPI between HNSCC tumor tissues and normal tissues (*p* < 0.01) (Figure 4A). With the survminer package and survival R package, patients with HNSCC were divided into high and low expression groups. Survival analysis showed that FPI was significantly associated with the OS of patients with HNSCC [*p* = 0.024, hazard ratio = 1.33, 95% confidence interval (CI) = 1.03–1.71], but not with PFI (*p* = 0.061, hazard ratio = 1.34, 95% CI = 0.96–1.89), or DSS (*p* = 0.108, hazard ratio = 1.64, 95% CI = 1–2.71) (Figure 4B).

A total of four differentially co-expressed genes, or hub genes, were identified based on the intersection of pseudotime analysis-derived differentially expressed genes, TCGA-derived differentially expressed genes, and FERROPTOSIS pathway genes: ACSL1, SLC39A14, TFRC, and PRNP (Figure 4C). The results of pseudotime analysis indicated that these four hub genes exhibited consistent expression changes in the differentiation process of HNSCC (Figure 4D).

### Correlation between the ferroptosis pathway and immunotherapy response

**FIGURE 5:**
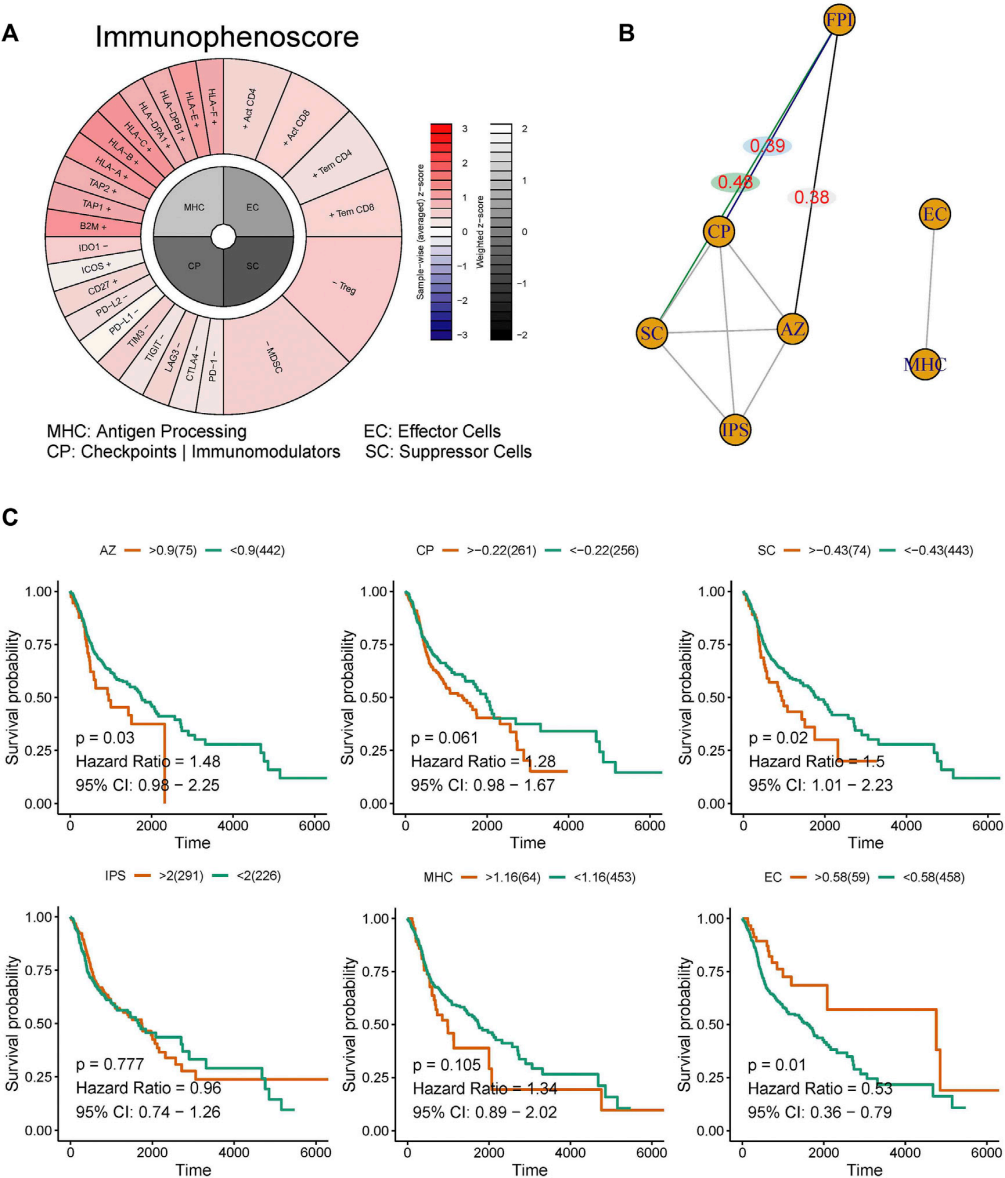
Correlation analysis among the ferroptosis pathway and immunotherapy response. **(A)** The immunophenotypes of tumor-immune landscape were calculated by Immunophenogram algorithm. Significant difference in FPI between HNSCC tumor tissues and normal tissues in TCGA. **(B)** The partial correlations based on spearman analysis were identified. **(C)** Survival analysis showed that AZ, SC, and EC were significantly associated with the OS of patients with HNSCC, but not with CP, MHC, and IPS.

The scores for WP-FERROPTOSIS and IPS were quantified (Figure 5A). Unsupervised clustering based on the correlation coefficients according to Spearman correlation analysis revealed close correlations between WP-FERROPTOSIS and antigen processing of MHC, effector cells (EC), immunomodulators of checkpoints (CP, correlation coefficient = 0.39, *p* = 0.04), suppressor cells (SC, correlation coefficient = 0.48, *p* = 0.03), and averaged Z (AZ, correlation coefficient = 0.38, *p* = 0.04) score (Figure 5B). Survival analysis showed that AZ [*p* = 0.03, hazard ratio = 1.48, 95% confidence interval (CI) = 0.98–2.25], SC (*p* = 0.02, hazard ratio = 1.50, 95% CI = 1.01–2.23), and EC (*p* = 0.01, hazard ratio = 0.53, 95% CI = 0.36–0.79) were significantly associated with the OS of patients with HNSCC, but not with CP (*p* = 0.06, hazard ratio = 1.28, 95% CI = 0.98–1.67), MHC (*p* = 0.11, hazard ratio = 1.34, 95% CI = 0.89–2.02), and IPS (*p* = 0.78, hazard ratio = 0.96, 95% CI = 0.74–1.26) (Figure 5C).

### Survival analysis and functional enrichment analysis of the hub genes

**FIGURE 6:**
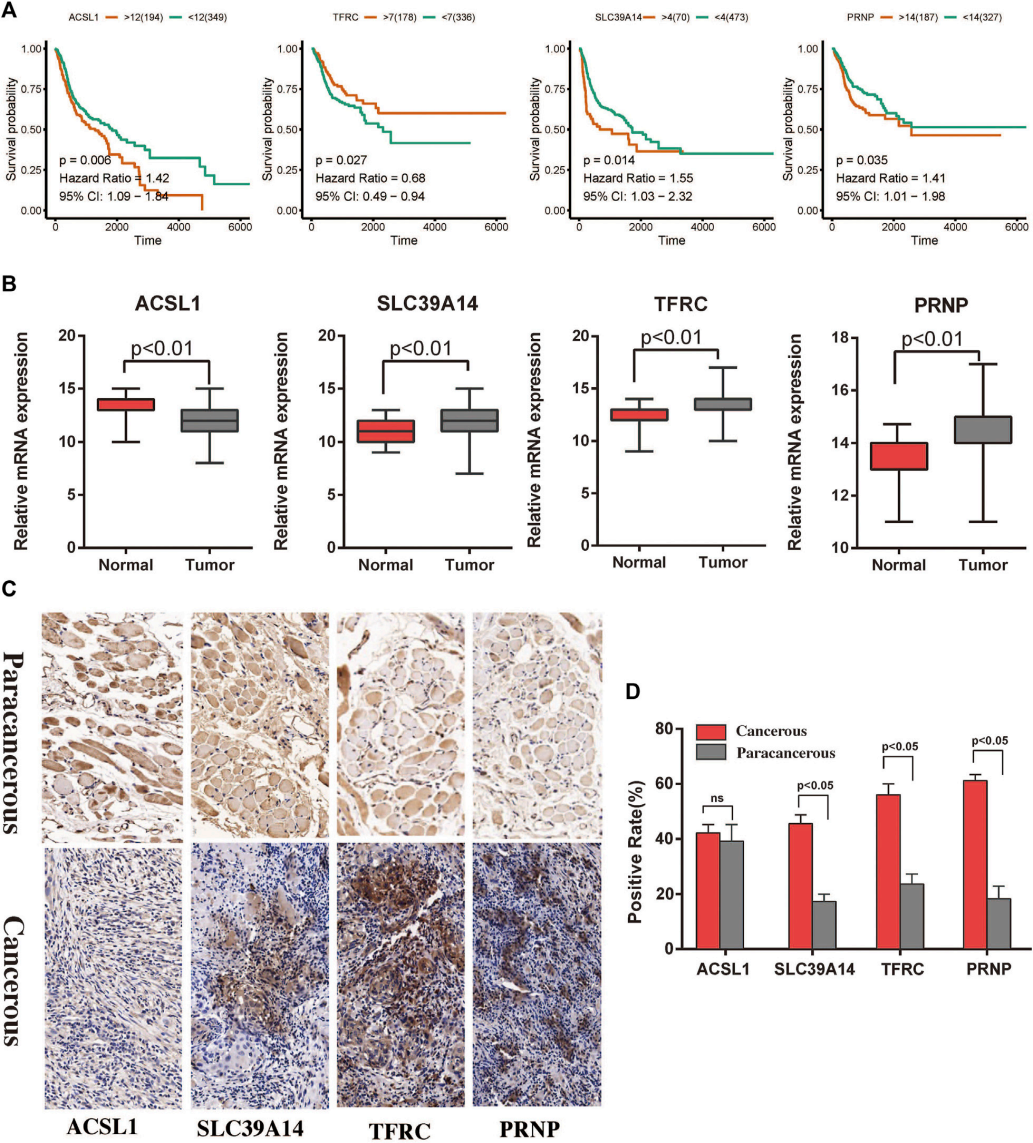
Survival analysis and immunohistochemistry to detect differential expression analysis of the hub genes. **(A)** Associations of the *ACSL1*, *SLC39A14*, *TFRC*, and *PRNP* genes with the survival of patients with HNSCC in TCGA. **(B)** Differential expression of the *ACSL1*, *SLC39A14*, *TFRC*, and *PRNP* genes between cancerous and paracancerous tissues in TCGA. **(C,D)** Expression of ferroptosis related proteins between the cancerous and paracancerous tissues (×400).

**FIGURE 7:**
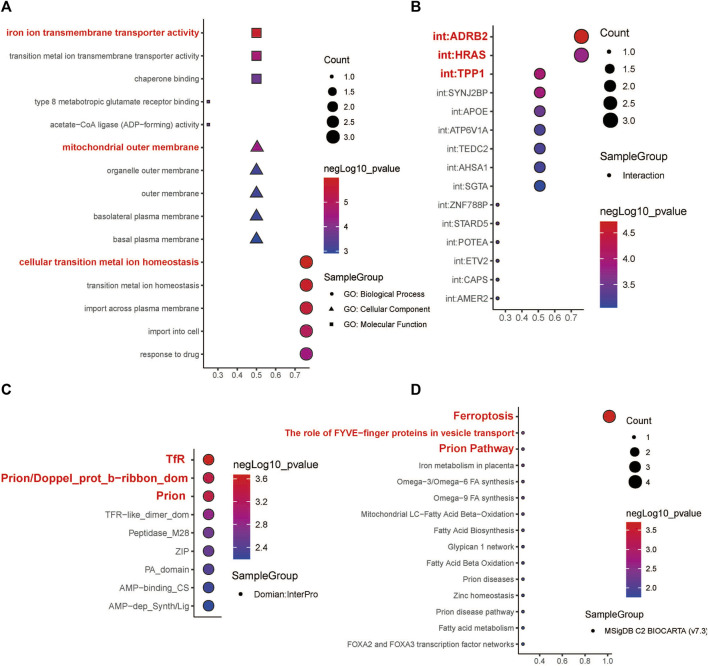
Functional enrichment analysis and molecular domain prediction of hub genes. **(A)** The biological process, cellular component, and molecular function that *ACSL1*, *SLC39A14*, *TFRC*, and *PRNP* were mainly involved in. **(B)** Possible interaction of *ACSL1*, *SLC39A14*, *TFRC*, and *PRNP* with *ADRB2*, *HRAS*, and *TPP1*. **(C)** Prediction of molecular domains of the hub genes. **(D)** Major molecular pathways through which the hub genes might participate in the progression of HNSCC.

The expression levels of the four hub genes were significantly associated with the survival of patients with HNSCC (Figure 6A). They also showed significantly different expression levels between the cancer tissues and the para-cancerous tissues of patients with HNSCC (Figure 6B). Our results indicated that SLC39A14, TFRC and PRNP are up-regulated in most HNSCC patients. However, there was no significant difference in the expression of ACSL1 (Figures 6C,D). The iron ion transmembrane transporter activity, mitochondrial outer membrane, and cellular transition metal ion homeostasis pathways were significantly enriched in the four hub gens (Figure 7A). Further, exploration of the biological mechanisms indicated that the hub genes may exert their effects in HNSCC through interactions with the *ADRB2*, *HRAS*, and *TPP1* genes (Figure 7B).

### Hub gene interaction and molecular domain prediction

According to the InterPro database, TfR, Prion/Doppel_prot_b-ribbon_dom, and Prion are likely to be the molecular domains of the four hub genes (Figure 7C). The four hub genes were mainly enriched in the following Biocarta pathways: Ferroptosis, The role of FYVE−finger proteins in vesicle transport, and Prion Pathway (Figure 7D).

## Discussion

The current study explores the role and mechanisms of the ferroptosis pathway in HNSCC, as ferroptosis is an important inhibitory mechanism in tumor cells. We believe that the findings shed light on the underlying ferroptosis-related mechanisms of HNSCC and can help in the identification of molecular markers that could accurately predict the outcome of HNSCC. These findings could eventually have immense clinical value for the treatment and prognosis of HNSCC.

Our results indicate that the ferroptosis pathway is closely associated with the overall survival of patients with HNSCC, and it is mainly involved in the regulation of extracellular matrix structure, humoral immune response, and vascular smooth muscle contraction. Additionally, it was found that ferroptosis pathway changes mainly occur in cancer stem cells, in comparison to other cell types; additionally, changes in the metal ion transport pathway were also significant in cancer cells. The latter finding supports the role of ferroptosis in HNSCC, as ferroptosis is induced by lipid ROS, which are largely generated by enzymes that contain iron or iron derivatives such as ferroheme and heme oxygenase-1.

According to the present findings, the expression levels of four genes, namely, *ACSL1*, *SLC39A14*, *TFRC*, and *PRNP*, were closely linked with ferroptosis, the occurrence of HNSCC, and the long-term outcome of patients. These genes are, therefore, potential targets for the development, progression, and treatment of HNSCC. Zhang et al. (2021) found that the higher expression level of ACSL1 could activates fatty-acid (FA) metabolic reprogramming during ovarian cancer metastasis and convert the lipid profile *via* AMP-activated protein kinase and Src pathways. Guo et al. (2020), demonstrated that the Acyl-CoA synthetase long-chain family member 1 (ACSL1) is an oncogene in thyroid cancers, and the higher expression level of ACSL1 led to a suppression of cancer cell progression and migration. Thomas et al. (2019), the activity of ACSL1 could convert the TNFα mediates inflammatory responses *via* attenuates the phosphorylation of p38 MAPK, ERK1/2, and NF-κB in breast cancer. Thorsen et al. (2011), illustrated that the alternative splicing of SLC39A14 is significantly difference among the adenomas and cancers, especially in the mutation of exclusive exons 4A, 4B, and the exon 4A/4B ratio. Wang et al. (2018), found that the expression level of SLC39A14 significantly correlated with the weightlessness of skeletal muscle mass and cachexia *via* regulated with zinc uptake in muscle progenitor cells. Xu et al. (2016), demonstrated that the lower expression level of SLC39A14 protein may significantly correlated with a higher Gleason score, advanced clinical stage, presence of metastasis, and prostate-specific antigen failure in human prostate cancer. Wu et al. (2019), reported that TFRC play a key role in cancer cellular metabolism and proto-oncogenic transcription *via* regulated by NF2-YAP signaling axis. TFRC regard as the key transporter in the intracellular iron, and the higher expression level of increased TFRC was associated with a worse prognosis for epithelial ovarian cancer patients (Huang et al., 2020). Stewart et al. (2019), shown that the expression level of TP63, PSAT1, and TFRC may correlated with the function of oxidation-reduction and glutathione in squamous cell lung cancer. Santos et al. (2021), found that the expression level of PRNP may significantly correlated with lymph node metastasis progression and worse prognosis for patients with head and neck squamous cell carcinoma. López-Cortés et al. (2020), demonstrated that the protein of PRNP, S100A9, DDA1, TXN, RPS27, S100A14, S100A7, MAPK1, AGR3 and NDUFA13 were considered as the best ranked metastasis driver proteins. AS an evolutionarily conserved cell surface protein, the higher expression level of PRNP closely related with the acquisition of malignant feature of cancer stem cells of multiple cancers, includes glioblastoma multiforme, breast cancer, and gastric cancer (Ryskalin et al., 2019).

In this study, the GSVA score for the ferroptosis pathway was closely correlated with the OS of patients with HNSCC, had a potential correlation with the PFI, and had no significant correlation with DSS. These findings indicate that HNSCC cells might escape ferroptosis through a balance of iron and thiol redox signaling. However, more research is required in the future to clarify the relationship between iron pathways, ferroptosis, and the origin of HNSCC.

## Conclusion

In summary, the ferroptosis pathway is closely associated with the development and progression of HNSCC, possibly through regulation of cancer stem cell proliferation. The *ACSL1*, *SLC39A14*, *TFRC*, and *PRNP* genes may be critical for ferroptosis-related development and progression of HNSCC, and may serve as potential treatment targets. These informative protein domains might be used in novel strategies for developing effective therapies targeting HNSCC. These findings also allow a better understanding of the molecular mechanisms involved in HNSCC development, thereby enabling the exploration of ferroptosis-related pathophysiology from a new perspective, and thus may raise current therapies to a new standard. Although these results are encouraging, we currently do not understand how hub regulators contributing to HNSCC development or which regulators with clinical transformation value, that improved prognostic outcomes. Thus, the further confirmed is required and necessary, based on animal models and large sample clinical verification, for the above analysis results.

## Data Availability

The datasets presented in this study can be found in online repositories. The names of the repository/repositories and accession number(s) can be found below: GEO with accession GSE103322.
